# Changes in the Heart Rate Variability in Patients with Obstructive Sleep Apnea and Its Response to Acute CPAP Treatment

**DOI:** 10.1371/journal.pone.0033769

**Published:** 2012-03-16

**Authors:** Ernesto Kufoy, Jose-Alberto Palma, Jon Lopez, Manuel Alegre, Elena Urrestarazu, Julio Artieda, Jorge Iriarte

**Affiliations:** 1 Clinical Neurophysiology Service, University Clinic of Navarra, Pamplona, Spain; 2 Department of Neurology, University Clinic of Navarra, Pamplona, Spain; 3 Neurophysiology Laboratory, Neurosciences Area, Centro de Investigación Médica Aplicada (CIMA), University of Navarra, Pamplona, Spain; University of Pennsylvania School of Medicine, United States of America

## Abstract

**Introduction:**

Obstructive Sleep Apnea (OSA) is a major risk factor for cardiovascular disease. The goal of this study was to demonstrate whether the use of CPAP produces significant changes in the heart rate or in the heart rate variability of patients with OSA in the first night of treatment and whether gender and obesity play a role in these differences.

**Methods:**

Single-center transversal study including patients with severe OSA corrected with CPAP. Only patients with total correction after CPAP were included. Patients underwent two sleep studies on consecutive nights: the first night a basal study, and the second with CPAP. We also analyzed the heart rate changes and their relationship with CPAP treatment, sleep stages, sex and body mass index. Twenty-minute segments of the ECG were selected from the sleep periods of REM, no-REM and awake. Heart rate (HR) and heart rate variability (HRV) were studied by comparing the R-R interval in the different conditions. We also compared samples from the basal study and CPAP nights.

**Results:**

39 patients (15 females, 24 males) were studied. The mean age was 50.67 years old, the mean AHI was 48.54, and mean body mass index was 33.41 kg/m^2^ (31.83 males, 35.95 females). Our results showed that HRV (SDNN) decreased after the use of CPAP during the first night of treatment, especially in non-REM sleep. Gender and obesity did not have any influence on our results.

**Conclusions:**

These findings support that cardiac variability improves as an acute effect, independently of gender or weight, in the first night of CPAP use in severe OSA patients, supporting the idea of continuous use and emphasizing that noncompliance of CPAP treatment should be avoided even if it is just once.

## Introduction

Obstructive Sleep Apnea (OSA) is considered a cardiovascular risk factor, which increases the likely of suffering stroke, congestive heart failure, arterial hypertension, pulmonary hypertension, coronary artery disease, cardiac arrhythmias, and acute myocardial infarction [1]–[6]. Although OSA has been associated with each of these diseases, the exact etiopathogenic mechanisms are unclear for most of these disorders. Among many of the systems affected by OSA, changes in the autonomic nervous system (ANS) are frequently studied [7], [8].

In healthy people, sleep stages have a great impact on sympathetic nervous system (SNS) activity, while the circadian system predominantly affects the parasympathetic nervous system (PNS) activity [9], [10]. Even people with altered sleep patterns (e.g. night shift workers) have an increased SNS activation [11]. In OSA patients, both sympathetic and parasympathetic nervous system control of the heart rate become unstable, with enhanced parasympathetic tone during the apneas and hypopneas punctuated with enhanced sympathetic nervous system activation subsequent to the apneic events [12], [13]. Although other cardiovascular diseases, such as hypertension (which is a common co-morbidity of OSA), are related to irregularities to both the PNS and SNS, dysregulation in these systems is noted during the daytime and nighttime in OSA patients even without evidence of cardiovascular diseases [14], [15].

Since OSA causes irregularities in the SNS function, OSA has been associated with an increased risk of cardiovascular events and mortality [16]. Besides, OSA severity is also linked to disturbances of PNS activity [17]. PNS activity is higher in non-REM sleep (NREM), particularly in stage 2 sleep, as compared with REM sleep. This increase in PNS activity during stage 2 can be seen in healthy subjects, but is even more noticeable in OSA, suggesting that increased parasympathetic activity is a consequence of mechanisms to compensate oxygen saturation (SatO_2_) fluctuations during NREM [18], [3].

One of the simplest non-invasive methods to monitor changes in cardiovascular control is by measuring heart rate variability (HRV), which can be defined as a physiological phenomenon where the time interval between heart beats varies. It is measured by the variation in the beat-to-beat interval. HRV reflects the relationship between the PNS and SNS, which is a good predictor of future cardiovascular problems [19]. HRV can be analyzed by using a time-domain analysis which measures beat to beat intervals (R-R, also known as NN intervals) [19]. The most commonly used time-domain HRV measure is SDNN (the standard deviation of all NN intervals). Changes and variability in heart rate are strongly influenced by sleep stages, and for some authors these changes do not differ significantly when comparing OSA and non-OSA patients [20]–[22]. R-R intervals have been shown to be at their highest in the early morning between the sleep and wake periods [23]. R-R intervals have also been shown to steadily increase from wake to non-REM sleep then decrease during REM sleep in healthy subjects [24]. Changes in the variances in R-R intervals have been associated with the severity of OSA [25], [26], although other recent studies have argued that spectral analysis of HRV may serve as a better method to determine of OSA severity [27].

Several studies have proposed the use of HRV as a simple and cheap solution for the diagnosis of OSA, but the limitations of current HRV analysis techniques and the multiple factors involved in OSA make it unlikely that HRV analysis can substitute polysomnogram (PSG) to diagnose OSA [28]–[31]

There exists evidence supporting the notion that CPAP treatment, which is a common therapeutic modality for OSA patients, affects HRV. Studies analyzing the effect of CPAP on HRV in healthy canines found that CPAP significantly increases cardiac output, heart rate and low frequency HRV (LF), while also finding a decrease in high frequency HRV (HF), showing that CPAP treatment can cause alterations in PNS and SNS in the absence of a cardiac or sleep breathing disorders. [32]. In healthy humans, no differences were found in PNS and SNS before or after CPAP treatment, but significant changes in PNS and SNS activity in non-apneic snorers were discovered [33], [34]. Another study showed an increased HRV after 1 month of CPAP treatment in patients with heart failure. This finding is interesting as low HRV is commonly seen in heart failure patients, and has been correlated with increased mortality [35]. Two long-term studies also showed improvement of HRV in OSA patient after CPAP treatment [36], [37]. While CPAP treatment improved HRV during sleep, the improvements in HRV did not last during the day [38].

Based on previous studies which showed a decreased HRV in patients with OSA and other cardiovascular disorders [34], [39], [40] using CPAP [41], our hypothesis it that very short-time period treatment with CPAP may improve HRV by correcting autonomic imbalance. Moreover, as patients with an increased body mass index (BMI) have a greater apnea hypopnea index (AHI), an increased amount of stage 1 sleep, decreased levels of SatO_2_ and, partially, a lower slow wave sleep time [42], obese patients may exhibit a different HRV pattern compared to those without obesity. In addition, several studies have shown that OSA is more common in men than women. Although the exact mechanisms are unclear, differences in obesity, anatomy and hormones are all though to play a role [43]. Thus, it is reasonable to hypothesise that HRV in patients with OSA may be different in men compared to women, as it has been shown that healthy women have a higher vagal tone than men [44], and whether there exist gender differences in the response to CPAP treatment [45].

According to hypothesis stated above, our specific aims in this study were: a) to HRV in patients with severe OSA; b) to determine the effectiveness of CPAP treatment for improving HRV in these patients in a very short time span (one night treatment); c) to determine if obesity and gender play a role in HRV in patients with severe OSA; and d) to discover if these changes and the effect of CPAP vary depending on the different sleep stages (REM, NREM, awake).

## Methods

This was a single-center transversal retrospective study involving patients with severe OSA (AHI>30). Subjects were selected from the sleep database of the Sleep Unit of the University Clinic of Navarra. Patients were either referred to the Sleep Unit from other departments within the University Clinic of Navarra (Neurology, Pulmonology, ENT, etc.) or from other hospitals of Spain. Most patients were referred because of primary complaints of snoring, daytime somnolence, restless sleep or other symptoms suggesting sleep apnea. Before PSG studies, all patients filled a questionnaire to determine clinical relevant data including current medications, current medical problems, alcohol, tobacco and recreational drugs consumption, Epworth scale, height and weight.

### Inclusion and exclusion criteria

Inclusion criteria included: (I) Severe OSA, defined as 30 or greater apneas and hypopneas per hour of sleep (AHI>30), (II) age range of 30–60 years old, (III) complete reversal of OSA during second PSG performed with CPAP treatment, and (IV) subjects who did not smoke, drink alcohol and did not consume recreational drugs.

Exclusion criteria included: (I) Atrial fibrillation and other cardiac arrhythmias; (II) myocardial ischemia, cardiomyopathy or myocardial infarction; (III) recent major surgery; (IV) cardiac pacemaker; (V) history of cerebrovascular disease; (VI) psychiatric disorders; (VII) other sleep disorders such as periodic limb movement disorder (PLMD), restless limb syndrome (RLS) or narcolepsy; (VIII) thyroid or other endocrine diseases, including diabetes mellitus; and (IX) treatment with antiarrhythmic, anticholinergic or antidepressant medications.

### Sleep Study, Measurements & CPAP Procedure

OSA was diagnosed based on a preliminary overnight PSG study. PSG studies were performed using Lamont amplifiers, 20 bit, 32 channels with 200 Hz sampling rates and dedicated inputs for EEG, single-lead ECG (lead II), tibial and chin EMG, oronasal flow, respiratory effort, oxymetry, heart rate and body position. CPAP machines were regular Resmed CPAP (California, USA).

Sleep stages, hypopneas, apneas and arousals were scored using the standard recommended American Academy of Sleep Medicine (AASM) scoring criteria [46]. Following the baseline study, subjects underwent a second PSG study to treat the OSA using the suitable CPAP pressure. The CPAP pressure was calculated according to neck circumference (NC), body mass index (BMI) and IAH, following the widely used equation previously described by Hoffstein et al: *P* = (0.16×BMI)+(0.13×NC)+(0.04+AHI)−5.12 [47] Only patients with total correction of apneas with CPAP were included. CPAP treatment was considered successful if AHI in the night with CPAP was less than 10 and the lowest oxygen value was higher than 89%.

### Sleep data conversion and HRV analysis

In order to assess changes in HRV from the patient's baseline study to their second study with CPAP treatment, 20-minute concatenated segments were selected from the sleep periods of REM, non-REM (NREM) and awake (WAKE), avoiding awakenings and artifacts. Although concatenated segments were taken, , an attempt was made to select consecutive or relatively close sleep stages in the selection process for REM and NREM segments, based on previous studies that show that HRV varies significantly throughout the night [19]. Most of the selected REM segments were not from the first quarter of sleep, but exceptions were made for certain CPAP studies which only consisted of one REM cycle. NREM segments were taken from stage 3 and stage 2 sleep and were excluded if they occurred in the first quarter of the night. Periods with ectopic cardiac beats and arousals were excluded from analysis.

Sleep data in the form of digital files were collected using the Stellate Reviewer software program. The sleep segments were saved as text files from their digital recording in Stellate Reviewer Version 6 (Stellate Inc., Montreal Canada), including the ECG, airflow and SaO2 signals. The text file containing the sleep data were converted into a Spike2 data file (S2R) using Spike 2 (version 6.02, Cambridge Electronic Design Limited, Cambridge UK). The S2R files were then analyzed using an HRV analysis program created through MATLAB (Mathworks Inc., Nattick Massachusetts, USA) by one of the authors (J.L.). Each ECG recording was manually inspected to avoid abnormal QRS wave morphology, movement artifacts, and to ensure that R-waves were correctly marked by the HRV analysis program to allow an accurate detection of R-R intervals.

The whole 20-minute segment was used to perform the analysis. The HRV program analyzed the data using a linear, time-domain analysis, which measures the mean and variance of the R-R intervals. The following measures were recorded from the HRV analysis program: a) Heart rate mean (R-R mean interval), b) HRV (measured as the standard deviation of NN intervals for the 20-minute segments [SDNN]), c) Minimal SatO_2_, d) Mean SatO_2_, e) Apnea duration, and f) Variance of SatO_2_ (Var. SatO_2_). Each of these 6 components was assessed during the selected sleep periods (WAKE, REM, NREM) in both the basal and CPAP study.

Comparisons of HRM, HRV and Var. SatO_2_ between the first (basal study) and the second night (with CPAP) were performed. Subgroups analysis comparing different sleep stages (WAKE, REM and NREM), obese versus non-obese, and male versus female were also performed.

### Statistical analysis

All statistical tests were performed using SPSS version 15.0.1 (SPSS Inc., Chicago, IL, USA). To investigate the effect of CPAP treatment by group, one-way ANOVA was performed. For categorical (qualitative) data, the χ2 test was used to check the differences between the two groups. When the expected frequencies were less than 5, Fisher's exact test was performed. For comparisons of two or more means, the analysis of variance (ANOVA) was performed. When a significant result was obtained in the ANOVA test, a *post-hoc* analysis (Scheffé's test) was carried out to perform multiple comparisons. In all cases, statistical significance was defined as *p*<0.05. A multivariate test was performed to test the effect of gender and obesity on CPAP treatment. To evaluate the changes between NREM and REM, a paired t-test was carried out.

### Standard protocol approval

This study was approved by the Institutional Review Board (IRB) of the University of Navarra. The IRB specifically waived the need for consent of participants, as this was a retrospective study. Data were anonymized by removal of direct identifiers from the data file (a variable was removed when it was highly identifying such as name, surname or place of birth; other variables which were irrelevant for analytical purposes were also removed).

## Results

### Sample characteristics

Thirty-nine patients (15 females, 24 males) with a mean age of 50.67 years (51.7 in females, 50 in males), a mean AHI of 48.54 (45.01 in females, 50.75 in males), a mean weight of 97.47 kg (100.5 in males, 92.52 in females), a mean height of 1.71 m (1.77 in males, 1.60 in females), and a mean body mass index (BMI) of 33.41 (31.83 in males, 35.95 in females) were included (**Table 1** **and** **2**). Comparisons between men and women revealed no statistical differences in age, severity of apneas, duration of apneas, number of oxygen desaturations per hour of sleep, minimal SatO_2_ and mean SatO_2_. Men were taller, and as the weight was similar, they tended to have lower BMI, but the difference was not significant. Twenty subjects were obese (BMI>30) and 6 had morbid obesity (BMI>35).

**Table 1:** Patients included in the study.

	N	Age ± SD (years)	Height ± SD (m)	Weight ± SD (kg)	BMI ± SD (kg/m^2^)
Male	24	50.70±6.74	1.77±.05	96.59±18.32	30.70±5.17
Female	15	51.73±8.33	1.60±.07	92.52±21.99	35.95±7.82
Total	39	51.40±7.35	1.70±.16	94.84±19.77	32.97±6.85

SD: Standard Deviation. BMI: Body Mass Index.

Men were taller than women. Women had a tendency to higher BMI but it was not significant. Age and AHI were similar.

**Table 2:** Respiratory features of patients included in the study.

	N	AHI ± SD	Apnea duration ± SD (s)	N° of oxygen desaturations per h of sleep ± SD	Minimal SatO_2_ ± SD (%)	Mean SatO_2_ ± SD (%)
Male	24	49.80±17.86	21.45±6.69	14.55±20.9	90.84±10.48	95.21±1.55
Female	15	45.01±10.36	18 ±4.86	17.18±14.94	88.86±8.8	94.85±1.07
Total	39	47.70±15.11	19.98±6.15	15.67±18.39	80±10.09	95.06±1.59

AHI: Apnea-Hypopnea Index. SD: Standard Deviation. SatO2: Variance of Oxygen Saturation.

### Relationship between HR parameters in OSA with CPAP treatment

The primary aim of this study was to determine how HRV in OSA patients is affected after acute CPAP treatment, in a single night of treatment. First we compared heart rate mean (HRM) and HRV, considering all the patients and stages together. With these data, no significant differences were seen in the HRM when comparing the basal study to the CPAP study (p>0.05). On the other hand, significant differences were seen when comparing HRV before and after CPAP treatment (*p*<0.05). As expected, significant differences did occur when comparing the variation in SatO_2_ levels before and after CPAP treatment (p<0.005). A summary of the results from this section is presented in **Table 3**.

**Table 3:** Differences between basal and CPAP studies in HRM, HRV and oxygen saturation.

Total	R-R interval (HRM)	HRV (ms)	Var. SatO_2_
Basal (first night)	0.9026±0.068 (66.4 bpm)	0.0673±0.011	1.1946±0.5338
CPAP (second night)	0.9282±0.073 (64.6 bpm)	0.0935±0.022	0.3857±0.1078
F	4.064	4.064	47.680
*p* value	0.229	0.045	<0.0001

CPAP: Continuous Positive Air Preassure. HRM: Heart Rate Mean. HRV: Heart Rate Variability. Var. SatO2: Variance of Oxygen Saturation.

### HRV in OSA and the different sleep stages

**Table 4** summarizes the results comparing HRM and HRV between the sleep stages during basal and CPAP studies. First we compared HRM and HRV in basal studies. HRM was higher in wakefulness, but it was similar in non-REM and REM (73 bpm vs. 64 bpm vs. 63 bpm, F = 7.598, p<0.005). In the CPAP studies, HRM was higher in wakefulness (73 vs. 63 vs. 61, F = 7.598, p<0.005). However, HRV was only significantly lower in the non-REM stage (0.490 ms) compared to wake (0.0837 ms) or REM sleep (0.0610 ms, T = 2.788, p<0.01).

**Table 4:** Heart rate mean and variability in OSA and sleep stages, in basal and CPAP studies.

	WAKE		Non-REM		REM	
	HRM	HRV (ms)	HRM	HRV (ms)	HRM	HRV (ms)
Basal	73*	0.0837	63	0.1060	64	0.0909
CPAP	71**	0.0919	62	0.0490***	62	0.0610
T	−0.656	−0.530	−0.944	2.283	1.565	1.565
*p* value	0.516	0.600	0.352	0.029	0.127	0.127

HRM: Heart Rate Mean. HRV: Heart Rate Variability.

Comparing basal and CPAP studies in the 3 situations, only HRV during non-REM was found to be lower in CPAP studies compared to basal studies. In the basal studies, HRM was higher in Wake (*) than in non-REM or REM. HRV was similar in the three situations. In the CPAP studies, HRM (**) was also higher in WAKE than in non-REM or REM. HRV was lower in non-REM (***), compared to REM and wakefulness.

### Relationship of HRV with obesity and gender

A multivariable analysis was performed in order to study the relationship between HRV and HRM and obesity and gender. Only HRV tended to be higher in obese patients (F = 3.848, p<0.05). No other differences were seen when comparing HRV parameters in all stages between obese and non-obese patients (p>0.05). **Table 5** shows how obesity did not play a significant role when comparing HRM or HRV during WAKE, NREM and REM stages. When differences between basal and CPAP studies were incorporated to the analysis, there were no differences in any of the parameters between basal and CPAP studies. When comparing the differences of HRV components in the basal study and CPAP study across the three sleep stages, no significant differences were seen between the obese and non-obese groups (p>0.05).

**Table 5:** Analysis of data in non-REM, REM and WAKE between obese and non-obese groups.

Obese vs. Non-obese	REM	NREM	WAKE
HRM	F = 3.294	F = 6.846	F = 3.472
	*p* = 0.07	*p* = 0.011*	*p* = 0.067
	T = 2.980	T = 0.464	T = −5.289
HRV	F = 3.848	F = 1.512	F = 0.48
	*p* = 0.052	*p* = 0.223	*p* = 0.490
	T = −4.897	T = −4.472	T = −4.840
Var. SatO_2_	F = 1.859	F = 1.350	F = 0.001
	*p* = 0.18	*p* = 0.250	*p* = 0.974
	T = 4.527	T = 5.472	T = 3.645

HRM: Heart Rate Mean. HRV: Heart Rate Variability. Var. SatO2: Variability in Oxygen Saturation.

* Statistical significance.

A multivariable analysis was also performed in order to notice whether any significant relationship existed between HRV and gender. The results are shown in **Table 6**. Globally, there were no differences (F = 1.877, p>0.06). Men had a higher HRM in all stages, and higher HRV only in WAKE. These results were similar in the basal and CPAP studies.

**Table 6:** Analysis of the data in non-REM, REM and WAKE between male and female groups.

Male vs. Female	REM	NREM	WAKE
HRM	F = 13.157	F = 5.021	F = 4.311
	*p* = 0.001*	*p* = 0.029*	*p* = 0,042*
	T = 1.540	T = 2.438	T = 1.262
HRV	F = 3.509	F = 3.426	F = 6.124
	*p* = 0.066	*p* = 0.069	*p* = 0.016*
	T = −0.954	T = −0.672	T = 2.785
Var. SatO_2_	F = 0.278	F = 0.001	F = 1.646
	*p* = 0.600	*p* = 0.972	*p* = 0.204
	T = 0.523	T = 0.657	T = 1.444

HRM: Heart Rate Mean. HRV: Heart Rate Variability. Var. SatO2: Variance of Oxygen Saturation.

* Statistical significance.

## Discussion

In this work, we aimed to study HRV during arousal-free sleep periods in a homogenous patient sample (free from medications, co-morbidities and any other sleep disorders) to analyze acute, very-short term, autonomic effects of CPAP treatment on severe OSA (AHI>30), and its differences depending on gender and obesity.

CPAP treatment has shown to improve HRV in OSA patients [36], [37], [48] and HRV is considered a more sensitive parameter than HRM to detect changes in ANS. Our results showed that, even in the first night with CPAP, the HRM was similar to the basal night but the HRV decreased significantly, considering all patients and sleep stages. Only patients with total normalization of the apnea index and oxygen values were included in the study; therefore, this result may be regarded as highly significant.

Given that, according to previous research, HRV is a good indicator of ANS activity, it is likely that CPAP treatment is able to reduce cardiac autonomic dysfunction in a very short time span. The results of this study also suggest that it may not be completely necessary for researchers and clinicians to wait months in order to see significant improvements in HRV.

Another of our aims was to see if the improvement of HRV was limited to either REM or NREM. According to our results, significant improvements in HRV were more relevant in NREM, even though some improvements were seen in REM sleep after CPAP treatment. A previous study suggests that increased PNS activity during NREM may be a compensating mechanism to SatO_2_ fluctuations, which REM sleep disrupts [18].

REM is a unique sleep stage from a physiological point of view when compared to NREM sleep stages because the heart rate and breathing rate are similar to WAKE. However, the results of this study did not support this vision, although our findings may be a consequence of the high severity of OSA and obesity seen in the patient group. The lack of significant changes in HRV in REM after CPAP treatment could be explained by the REM interference theory in PNS activity. The clinical implication of these results may suggest that patients who suffer from OSA with apneas occurring predominantly during REM sleep, may not enjoy the same cardiovascular benefits compared to OSA patients whose apneic episodes are scattered between REM and NREM. However, further studies are required to determine whether the unique properties of REM sleep influence autonomic function in patients with OSA.

The heart rate mean did not decrease significantly from NREM to REM during CPAP studies. This result is similar to previous studies in healthy individuals [24]. The decrease HRV from NREM to REM was seen in basal studies, which suggests that OSA may influence the variance of the heart rate, but does not vary the natural change of overall beats per minute from NREM to REM. The lack of change in HRM during the CPAP study suggests that CPAP treatment lowers the variance of heart rate during NREM, as well as eliminates the change in the heart rate between NREM and REM.

Previous studies showed obese patients to have lower variations in total HRV [49]. Our results also showed significant differences in HRV in obese patients during REM when compared to the non-obese group in both CPAP and basal studies. From these results, it seems plausible that CPAP treatment may improve HRV in obese patients, sufficiently so as to resemble the improved HRV seen in non-obese patients with CPAP treatment. In our study, another aim was to study the effects of obesity on CPAP treatment in severe OSA (AHI>30) patients, as CPAP treatment has shown to lower night time blood pressure and increase SNS activity in OSA patients [50]. The results from our study showed that obesity does not influence HRV or HRM during CPAP treatment when compared to non-obese patients. Based on these results, obesity does seem to influence heart rate mean in WAKE and REM, but not enough to be statistically significant. Therefore, from a clinical perspective, additional or differential forms of treatment do not seem necessary when combating ANS dysfunction in obese patients.

The final aim of our study was to determine the role of gender in HRV during short-term CPAP treatment of severe OSA (AHI>30) patients, as women with breathing disorders have been shown to have increased SNS activity during NREM, and men with breathing disorders have been shown to have lower PNS activity during wake [51], and women with high AHI had low SNS activation during REM [52]. Our results did show a difference in HRV between men and women in wakefulness. In addition, HRM was different in all the sleep stages. Our results showed a significant difference in HRV and heart rate mean between our male and female groups during wake. However, the changes were similar in the basal and in the CPAP nights, which suggests that gender does not influence the improvements seen in ANS activity from CPAP treatment. Nevertheless, the absence of statistical significance when assessing the effects of gender and obesity on HRV may be a consequence of the small sample size of our study, which is one of its limitations.

The main limitation of our study is a consequence of its transversal nature. In these regard, there is a possibility that changes in HRV during acute CPAP treatment may reflect a normalization of the respiratory pattern or may be due to changes in venous return secondary to the dramatic changes of intrathoracic pressure that occur during the apneic event rather than changes in cardiovascular control mechanisms. Thus, caution must be used when analyzing our results, as the effect of OSA in autonomic activity may perhaps more likely to be detected by comparing HRV on the first night of treatment with a sleep study on CPAP after weeks of treatment, and not with a single-night study, such as the present one. We neither studied whether the changes in HRV were associated with the severity of OSA, as we only selected patients with an AHI>30. Moreover, we do not provide data regarding the percentage of stage 2 and stage 3 sleep in the NREM segments, in spite of the fact that several studies show that ANS balance differs between these 2 stages. Finally, we did not study frequency-domain measures (i.e. HF, LF, and VLF); hence, further studies may be needed to understand our findings in depth.

Nonetheless, all these limitations do not hamper the fact that cardiac variability improves as an acute effect, independently of gender or weight, in the first night of CPAP use in severe OSA patients. Thus, we think that the CPAP treatment should not be delayed. Severe OSA patients should be advised that even a single night without CPAP has changes in the cardiac rate, which are corrected with CPAP. Hence, to use or not to use the CPAP for a single night does matter.
